# Surgical management of retrosternal thyroid disease: a decennial retrospective analysis

**DOI:** 10.3389/fendo.2026.1739552

**Published:** 2026-02-27

**Authors:** Francesco Quaglino, Domenico Galetta, Luca Cestino, Federico Festa, Sara Ruscio, Giulia Carbonaro, Marta Breda, Giorgia Gavello, Samuela Cannazza

**Affiliations:** 1 General Surgery Division, Maria Vittoria Hospital, Azienda Sanitaria Locale (ASL) Città di Torino, Turin, Italy; 2 Thoracic Surgery Division, San Giovanni Bosco Hospital, Azienda Sanitaria Locale (ASL) Città di Torino, Turin, Italy

**Keywords:** cervical surgical approach, hypocalcaemia, Imaging-based classification, predictive factors, recurrent laryngeal nerve palsy, retrosternal thyroid disease

## Abstract

**Introduction:**

Thyroid disease with retrosternal extension lacks a universally accepted definition in the literature, contributing to significant variability in the reported prevalence across series. This study aims to present the experience of a high-volume center in the management of retrosternal thyroid pathology and to identify potential predictive factors that may assist in surgical planning.

**Materials and methods:**

Between 1 January 2014 and 31 December 2024 we performed 1347 thyroidectomies: 355 (26,3%) thyroid surgical procedures were performed in patients whose thyroid pathology was identified intra-operatively as retrosternal extension. We analyzed patient comorbidities, any pre-operative treatments, pre-operative imaging, the type of surgical procedure performed, the surgical approach adopted, and post-operative complications. Four definitions of retrosternal thyroid pathology, selected among those most frequently cited in the literature and deemed applicable in clinical practice, were compared.

**Results:**

Benign retrosternal goiter accounted for 84.2% of cases, thyroid malignancies represented 6.2% of the cohort, while retrosternal goiters with indeterminate cytology comprised 9.6% of the total. Total thyroidectomy was performed in 80% of patients. Among the 355 patients operated on, a classical cervical approach (Kocher incision, optionally with split cutaneous layer) was used in 99.2% of cases; only 0.8% required an additional thoracic approach. Pre-operative diagnosis of malignancy and the type of surgical procedure performed emerged as the principal factors associated with the development of recurrent laryngeal nerve palsy and hypocalcaemia in the postoperative period.

**Conclusions:**

A universally recognized definition of retrosternal thyroid pathology remains elusive. In high-volume centers the cervical approach is sufficient and the rates of thoracic surgical approach is less than 1%. Computed tomography (CT) or magnetic resonance imaging (MRI) of the cervical region are the modalities of choice for investigating the retrosternal component of the thyroid, as they allow accurate assessment of mediastinal extension and facilitate surgical planning. In our study, the integration of the Huins classification and the Cohen & Cho classification proved to be a valid predictive factor for identifying cases that may require a surgical approach beyond the classical Kocher-type cervicotomy. We recommend that retrosternal thyroid pathologies be managed in referral centers equipped with multidisciplinary expertise and adequate surgical experience to ensure optimal outcomes.

## Introduction

Thyroid pathologies that extend below the thoracic inlet lack a standardized definition, and clear guidelines for preoperatively identifying patients who may require a thoracic approach are still unavailable (1). Multinodular goiter (MNG) is the thyroid condition most frequently associated with retrosternal extension and represents the most common indication for surgical intervention. MNG may enlarge from the cervical region into the mediastinum through the thoracic inlet, or, in rare cases, ectopic thyroid tissue may develop directly within the mediastinum (2).

Numerous definitions exist in the literature to describe this clinical condition. Although similar, these definitions do not completely overlap, which contributes to the wide variability in reported incidence, ranging from 12% to 46% (3, 4). The definitions most frequently cited, and those most applicable in clinical practice due to their reliance on second-level imaging such as cervical CT or MRI, are as follows.

Katlic et al. defined a retrosternal thyroid gland as one in which more than 50% of its volume extends into the retrosternal space. In a related definition, a substernal goiter is described as a thyroid gland located partially or entirely within the mediastinum, descending at least 3 cm below the sternal manubrium or two fingerbreadths below the thoracic inlet in the operative position (5).

Huins et al. proposed a three-grade classification system based on the relationship between retrosternal thyroid tissue and the aortic arch: grade 1, the tissue lies above the aortic arch; grade 2, it is positioned between the aortic arch and the pericardium; and grade 3, the gland extends below the right atrium (1). Cohen and Cho categorized retrosternal thyroid pathology into four grades according to the proportion of thyroid tissue present in the mediastinum: grade 1 (<25%), grade 2 (26–50%), grade 3 (51–75%), and grade 4 (>75%) (6).

Ultrasound remains the primary tool for morphological assessment of the thyroid; however, its evaluation of retrosternal portions of substernal goiters can be challenging (7, 8). Consequently, patients with retrosternal thyroid disease are typically assessed using additional imaging modalities, including posteroanterior chest radiography, computed tomography (CT), and magnetic resonance imaging (MRI) (9). CT is particularly recommended for preoperative planning, as it provides detailed information on thyroid morphology, size, mediastinal extension, and anatomical relationships (10–12).

Total thyroidectomy with en bloc removal of the intrathoracic component remains the definitive treatment for retrosternal thyroid disease. Literature indicates that approximately 95% of intrathoracic goiters can be removed via a cervical approach, while the remaining 5% require an extracervical approach, tailored to the goiter’s extent and position (11, 13, 14). Surgery for retrosternal thyroid disease carries higher complication rates compared to standard cervical thyroidectomy, including postoperative bleeding, transient and permanent unilateral or bilateral recurrent laryngeal nerve injury, and transient or permanent hypoparathyroidism (12, 13, 15).

This study presents the experience of the Reference Center for Thyroid and Endocrine Gland Neoplasms, Regional Oncology Network, Maria Vittoria Hospital, Turin, in the management of retrosternal thyroid disease. We aimed to identify predictive factors that could support surgeons in preoperative planning. To this end, we conducted a retrospective analysis of 355 patients with confirmed retrosternal thyroid pathology, as determined intraoperatively. Patient comorbidities, preoperative treatments, imaging (primarily cervical CT/MRI), type of surgery, surgical approach, and postoperative complications were systematically evaluated.

## Materials and methods

This study analyzed all thyroid surgeries performed between January 1, 2014, and December 31, 2024, totaling 1,347 procedures. From this cohort, patients with intraoperative evidence of retrosternal thyroid pathology, as identified by the operating surgeon, were selected for inclusion.

All surgical procedures were performed by experienced endocrine surgeons working in a high-volume referral center for thyroid disease. This specialized context reduces intraoperative variability and ensures a high level of expertise in identifying retrosternal characteristics. Furthermore, all patients underwent a preoperative evaluation including an endocrine surgical consultation and cervical ultrasound, which allowed for accurate selection and documentation of the anatomical presentation prior to surgery.

The retrospective analysis comprises 355 patients (238 women and 117 men), aged 18 to 88 years, with a mean age of approximately 60 years in both sexes.

All surgeries were conducted by experienced endocrine surgeons of the Complex Operative Unit of General Surgery 1 at Maria Vittoria Hospital, Turin, under the direction of Dr. Francesco Quaglino. The center is a Reference Center for Thyroid Neoplastic Pathology within the Piemonte Oncology Network and is accredited by the Italian Society of Endocrine Surgery.

The study included patients with both benign and malignant thyroid pathologies, with preoperative diagnoses classified into five categories: thyroid goiter (multinodular, uninodular, or with a cytologically TIR1/TIR2 nodule), thyroid goiter with indeterminate lesion (TIR3A/TIR3B nodule), isolated indeterminate lesion (TIR3A/TIR3B), thyroid neoplasia (TIR4/TIR5), and Graves’ disease.

Surgical procedures performed included lobectomy with isthmectomy (LIT), total thyroidectomy (TT), thyroid completion surgery—performed in patients previously submitted to subtotal thyroidectomy or lobectomy, in cases of retrosternal goiter recurrence or detection of neoplasia on definitive histology—and, in one case, thyroid biopsy.

The surgical approaches analyzed included classical Kocher cervicotomy, cervicotomy with skin split, sternotomy, and posterolateral thoracotomy.

For the purpose of this study, namely to identify potential predictive factors for the need to perform additional surgical maneuvers beyond the standard Kocher cervicotomy, we chose to include the skin split among the extracervical maneuvers. This decision was made because, despite being a relatively minor extension, the skin split is still a surgical step performed to widen the operative field and therefore represents a deviation from the classical cervicotomy alone.

The most relevant postoperative complications considered were:

• Postoperative hypocalcemia, defined as serum calcium corrected for albumin <2.00 mmol/L, or the need for replacement therapy and/or the presence of clinical symptoms requiring treatment. Hypocalcemia was considered permanent if therapy discontinuation was not possible beyond six months post-surgery (12). PTH measurement is not routinely requested in our practice for patients undergoing surgery for thyroid disease.

• Recurrent Laryngeal Nerve Palsy

Unilateral or bilateral recurrent laryngeal nerve palsy, whether transient or permanent, diagnosed via postoperative laryngoscopy; considered permanent if persistent 12 months post-surgery (16). For all patients is performed a post-operative laryngoscopy.

• Reoperation Due to Postoperative Hemorrhage

Reintervention required for postoperative bleeding.

In our center, intraoperative neuromonitoring is employed for all patients undergoing total thyroidectomy, as well as for pediatric patients and in cases of reoperation.

Data were collected using various software tools and methodologies, subsequently processed to yield schematic classifications amenable to statistical analysis. We used the Chi-square test and the statistical significance threshold (P-value) was set at 0.05.

## Results

Between January 1, 2014, and December 31, 2024, the General Surgery Department at Maria Vittoria Hospital performed 1,347 thyroid surgeries. Of these, 355 cases (26%) were intraoperatively identified as having a retrosternal thyroid, comprising 238 females and 117 males, aged 18 to 88 years (mean age approximately 60 years).

Preoperative diagnosis revealed that 84.2% (299 patients) had a goiter, while 6.2% (22 patients) were diagnosed with neoplasia (**Table 1**).

**Table 1:** Preoperative diagnosis.

Preoperative diagnosis	Total	Percentage
GRAVE'S DISEASE	20	5.6%
INDETERMINATE LESION	7	2.0%
THYROID NEOPLASIA	22	6.2%
GOITER (STRUMA)	299	84.2%
GOITER WITH INDETERMINATE LESION	7	2.0%
TOTAL	**355**	**100%**

Distribution of clinical-pathological diagnoses in the study cohort (n = 355).

Boldface is used to indicate the total values.

Surgical procedures included 284 total thyroidectomies (80%), 52 lobectomy-isthmectomies, 18 completion thyroidectomies, and one thyroid biopsy.

Notably, one patient undergoing completion thyroidectomy for goiter was found to have papillary thyroid carcinoma upon final histopathological examination. Subsequent staging CT revealed ectopic retrosternal thyroid tissue in the mediastinum, leading to reoperation two months later.

The thyroid biopsy was performed on a patient who was initially scheduled for a total thyroidectomy based on a preoperative fine-needle aspiration result of TIR5, indicating a high suspicion of malignancy. However, intraoperatively the patient was found to have a much more advanced oncologic scenario than suggested by the preoperative staging diagnostics, making it not feasible to perform an oncologically radical surgical procedure at that time. Therefore, only a thyroid biopsy was carried out to obtain a definitive histological diagnosis.

**Figure 1:**
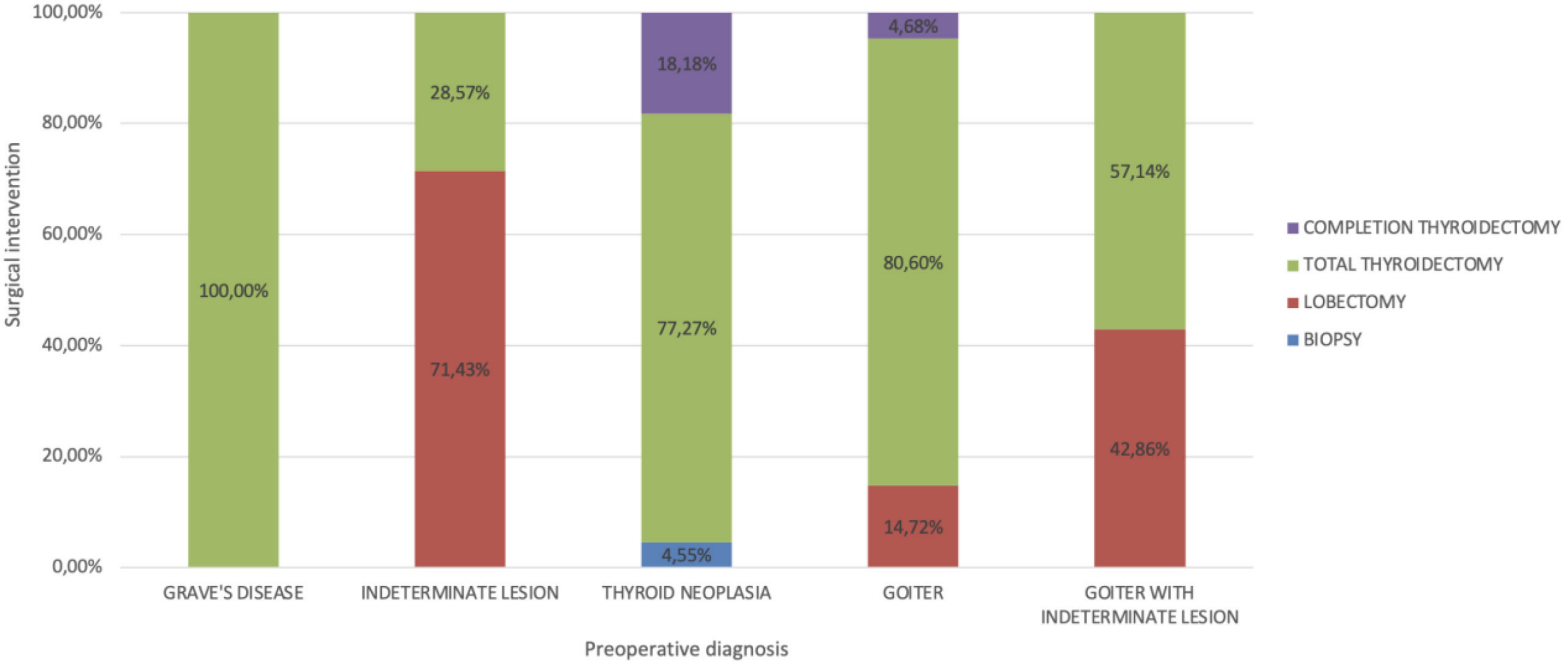
Correlation between preoperative diagnosis and type of surgical intervention performed.

**Table 2** presents the surgical approaches employed during the study period. **Figure 1** illustrates the correlation between preoperative diagnosis and the type of surgical intervention performed. Notably, 241 (80,6%) total thyroidectomies were conducted for patients diagnosed with multinodular goiter.

**Table 2:** Surgical approach.

Surgical approach	Total	Percentage
CERVICOTOMY	317	89.3%
CERVICOTOMY WITH SKIN-SPLIT INCISION	35	9.9%
STERNOTOMY	2	0.6%
POSTEROLATERAL THORACOTOMY	1	0.3%
TOTAL	**355**	**100%**

Surgical approaches employed.

Boldface is used to indicate the total values.

**Figure 2:**
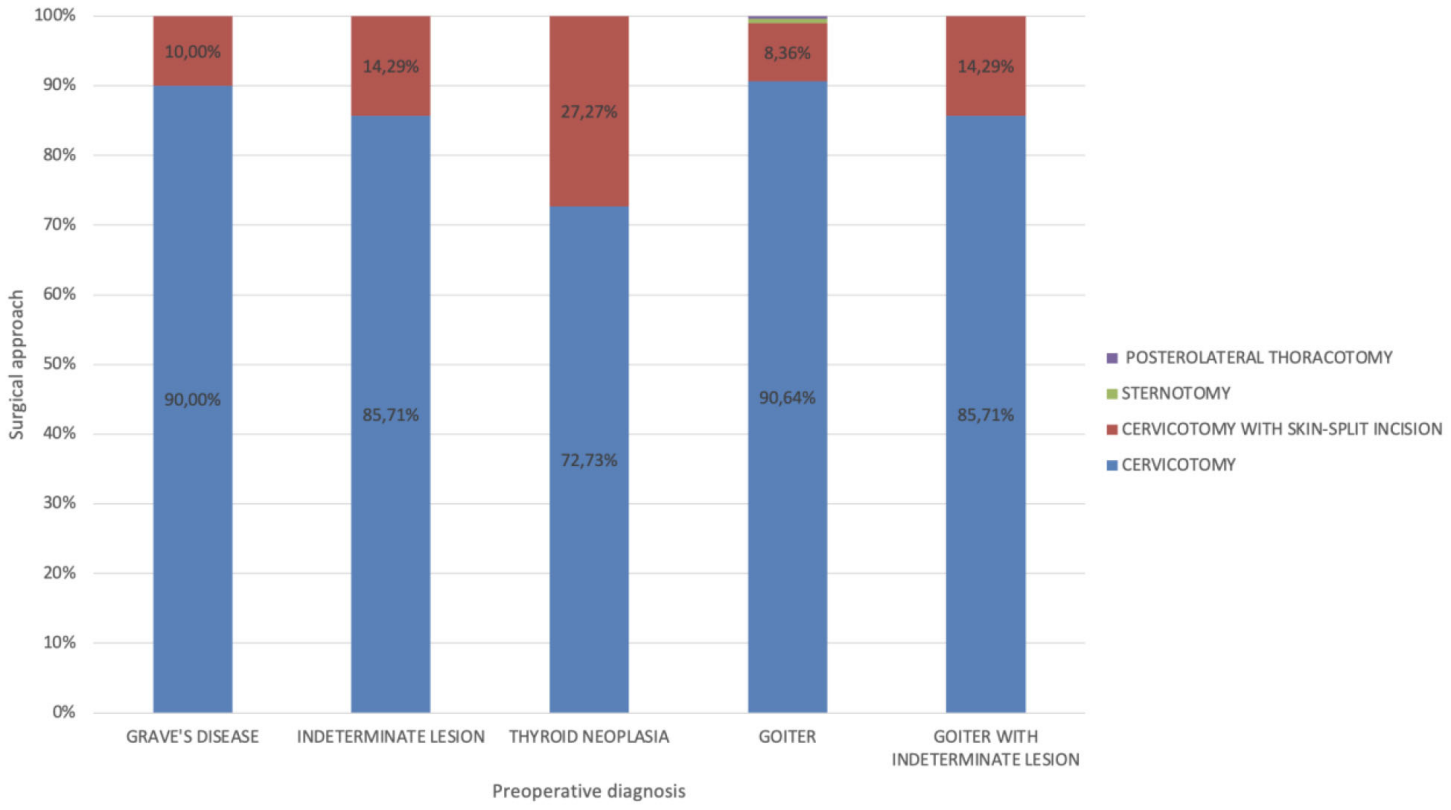
Correlation between preoperative diagnosis and surgical approach utilized.

**Figure 2** depicts the relationship between preoperative diagnosis and the surgical approach utilized in the operating room. The data indicate that patients with thyroid neoplasia required more complex surgical maneuvers beyond the standard Kocher cervical incision. Specifically, a split cutaneous incision was necessary in 27.3% of neoplastic cases, compared to an overall average of 9.86% for all procedures.

For 128 of the 355 patients, we had available radiological images, specifically CT or MRI of the cervical region. This discrepancy can be explained on the one hand by the fact that in recent years there has been an increased utilization of second-level imaging for retrosternal thyroid pathology, rising from an average of 23 % in the first six years to 51.2 % in the last five years. On the other hand, some patients had undergone these examinations outside of our center, and it was therefore not possible to retrieve these images for inclusion in the study.

**FIGURE 3:**
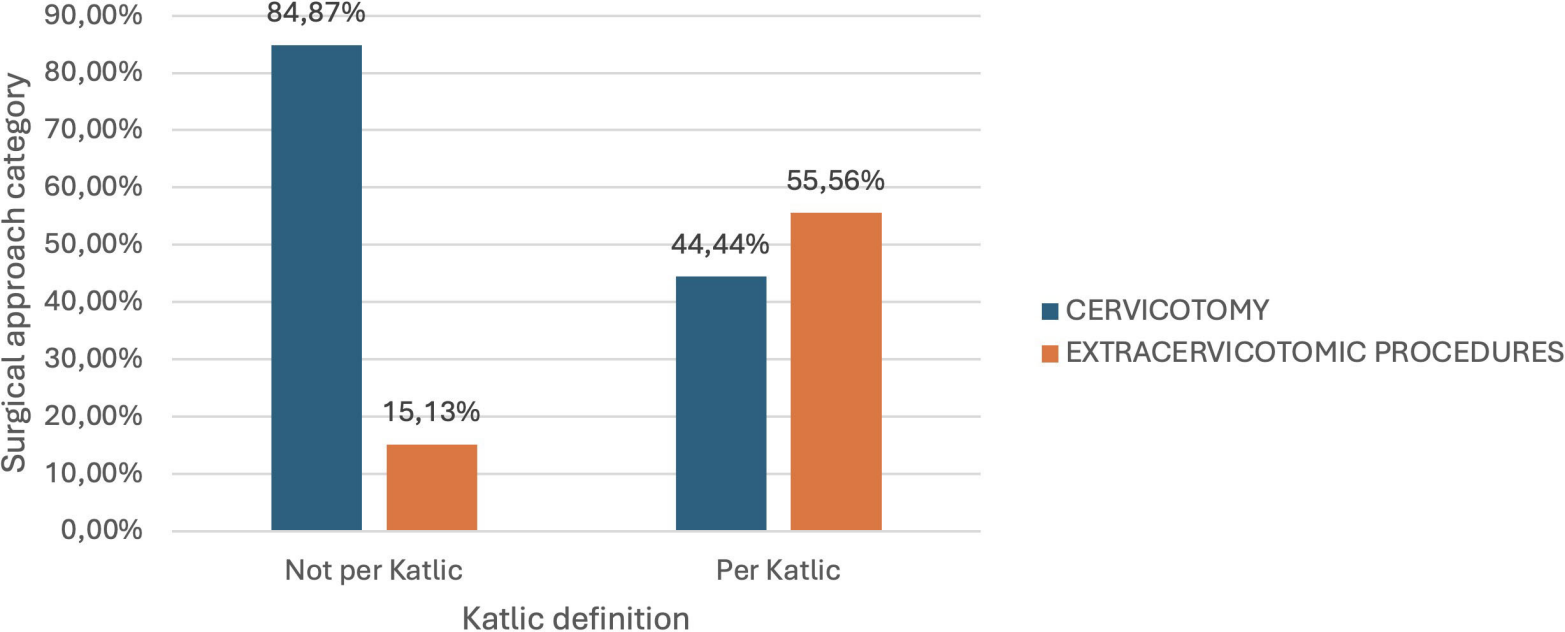
Correlation between surgical approach and Katlic classification of substernal goiter.

**FIGURE 4:**
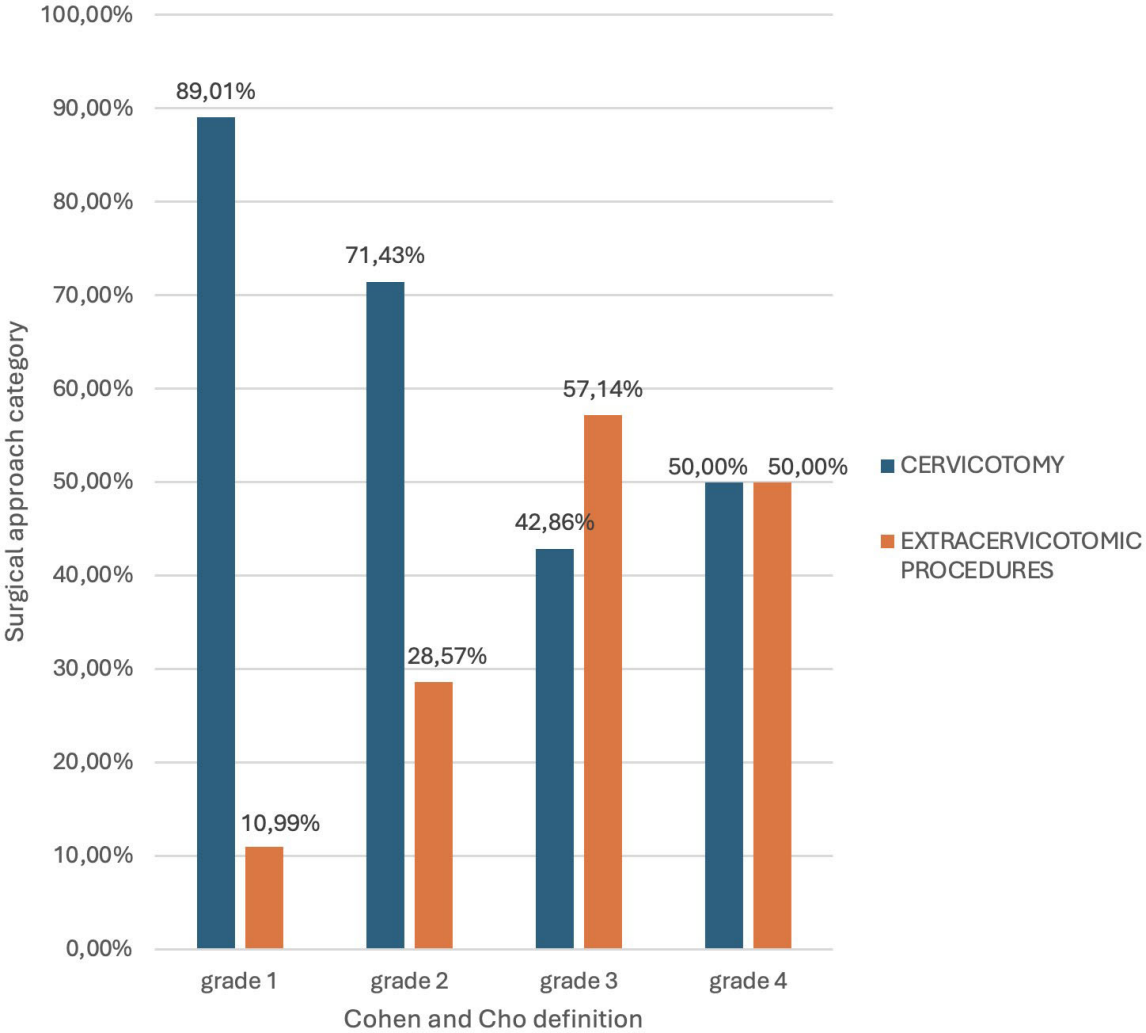
Correlation between surgical approach and the Cohen & Cho classification (stratified according to the percentage of thyroid gland mass located within the mediastinum).

**FIGURE 5:**
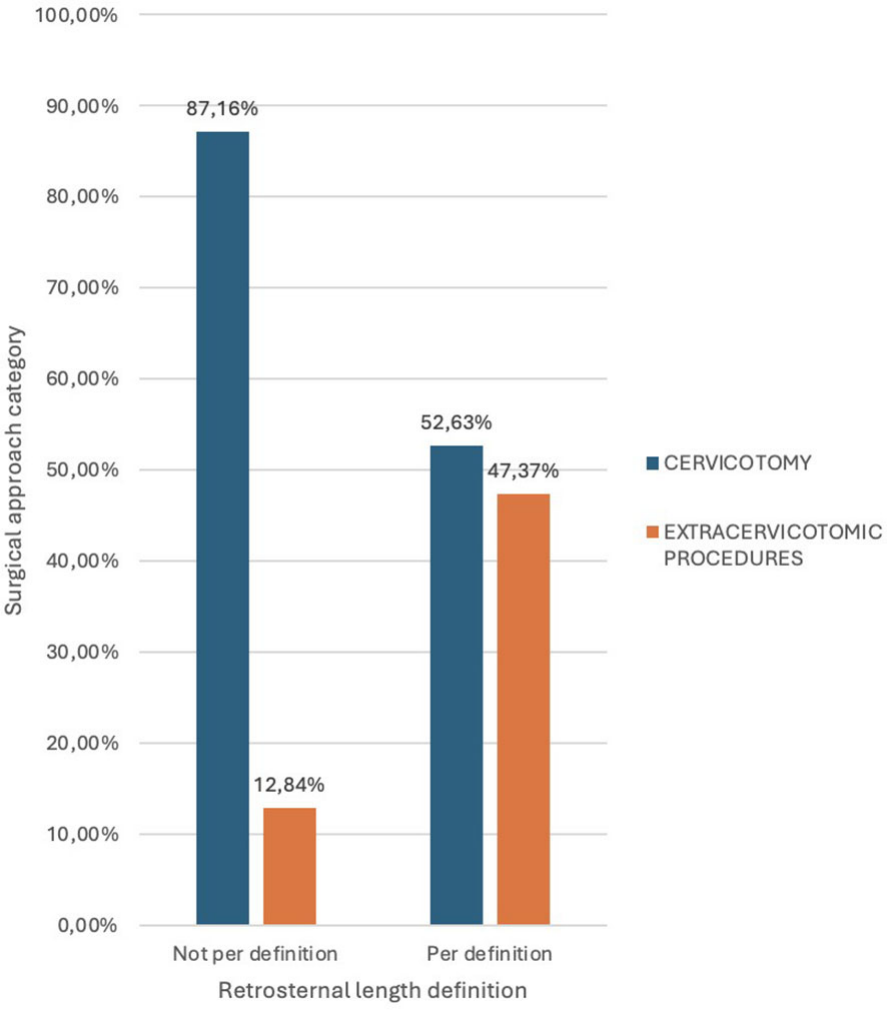
Correlation between surgical approach and definition based on retrosternal length (extension of at least 3 cm behind the sternum).

**FIGURE 6:**
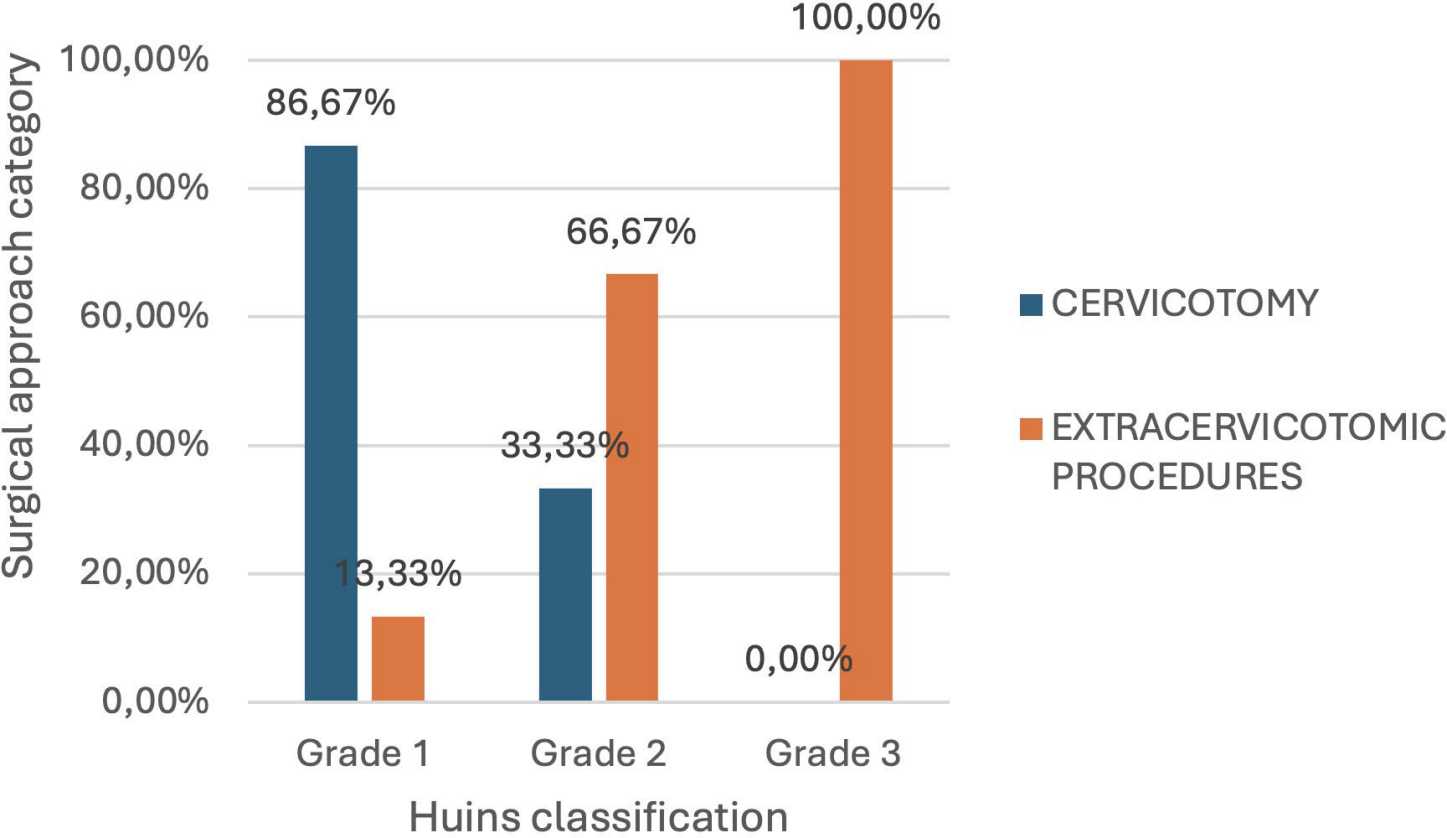
Correlation between surgical approach and the Huins classification (based on the relationship between retrosternal extension of the thyroid gland and the aortic arch/right atrium).

These 128 patients were subsequently categorized according to the four definitions utilized in this study. The following graphs (**Figures 3**–**6**) illustrate that all patients falling within these definitions exhibited a statistically significant increased need for additional surgical procedures compared to the standard Kocher cervicalotomy, when compared to those whose condition did not meet these definitions.

Regarding complications, 94.08% of patients did not experience recurrent laryngeal nerve (RLN) palsy. Transient unilateral RLN palsy occurred in 5.07% of cases, while transient bilateral RLN palsy was observed in 0.56% of patients (2 cases), one of whom required temporary tracheostomy with complete recovery after two months. In two patients, RLN sacrifice was necessary for oncological radicality. One patient (0.28%) sustained permanent unilateral RLN palsy due to intraoperative nerve injury, which was recognized and addressed during the procedure.

In 74.08% of cases, no postoperative hypocalcemia occurred; transient hypocalcemia was observed in 22.25% of cases, while definitive hypocalcemia was noted in 3.66%. These complications were correlated with preoperative diagnosis and the type of surgical intervention performed. The data revealed that the most significant factors influencing these complications were a preoperative diagnosis of neoplasia and the performance of total or completion thyroidectomy. The surgical approach itself did not impact the development of these complications.

Specifically, in patients with neoplasia, the incidence of transient unilateral recurrent laryngeal nerve (RLN) palsy was 18.18% (4 cases), compared to an overall average of 5.07% (18 cases). This difference was statistically significant (p-value = 0.03). Completion thyroidectomy procedures exhibited a 16.7% incidence of transient unilateral RLN palsy, compared to 4.6% in total thyroidectomies and 3.8% in lobectomies. Again, the type of surgery was significantly associated with the occurrence of this complication (p-value = 0.02) (**Tables 3**, **4**).

**Table 3:** Correlation between preoperative diagnosis and recurrent laryngeal nerve paralysis.

Preoperative diagnosis						
Complications	Grave's disease	Indeterminate lesion	Thyroid neoplasia	Goiter	Goiter with indeterminate lesion	Total
No lesion	95.00%	100.00%	81.82%	94.98%	85.71%	94.08%
Intraoperative ricorrent lesion	0.00%	0.00%	0.00%	0.33%	0.00%	0.28%
Transient unilateral recurrent nerve paralysis	5.00%	0.00%	18.18%	4.01%	14.29%	5.07%
Transient bilateral recurrent nerve paralysis	0.00%	0.00%	0.00%	0.67%	0.00%	0.56%
Total	100%	100%	100%	100%	100%	100%

**Table 4:** Correlation between type of surgical intervention and recurrent laryngeal nerve paralysis.

Surgical intervention				
Complications	Lobectomy	Total thyroidectomy	Completion thyroidectomy	Total
No lesion	94.23%	94.72%	83.33%	94.08%
Intraoperative ricorrent lesion	1.92%	0.00%	0.00%	0.28%
Transient unilateral recurrent nerve paralysis	3.85%	4.58%	16.67%	5.07%
Transient bilateral recurrent nerve paralysis	0.00%	0.70%	0.00%	0.56%
Total	100%	100%	100%	100%

The incidence of transient hypocalcemia among patients with a preoperative diagnosis of neoplasia was 36%, compared to an overall average of 22.3%. Total and completion thyroidectomy procedures were associated with a significantly higher risk of postoperative hypocalcemia, with transient hypocalcemia occurring in approximately 26% of cases and definitive hypocalcemia in 4%. This risk was notably higher compared to patients undergoing lobectomy-isthmectomy, in whom the incidence of hypocalcemia was 1.92% (p-value = 0.009) (**Tables 5**, **6**).

**Table 5:** Correlation between preoperative diagnosis and postoperative hypocalcemia.

Preoperative diagnosis						Total
Complications	Grave's disease	Indeterminate lesion	Thyroid neoplasia	Goiter	Goiter with indeterminate lesion	
No hypocalcemia	75.00%	85.71%	63.64%	73.91%	100.00%	74.08%
Transient hypocalcemia	20.00%	14.29%	36.36%	22.07%	0.00%	22.25%
Permanent hypocalcemia	5.00%	0.00%	0.00%	4.01%	0.00%	3.66%
Total	100%	100%	100%	100%	100%	100%

**Table 6:** Correlation between surgical intervention and postoperative hypocalcemia.

Surgical intervention				Total
Complications	Lobectomy	Total thyroidectomy	Completion thyroidectomy	
No hypocalcemia	98.08%	70.07%	66.67%	74.08%
Transient hypocalcemia	1.92%	25.70%	27.78%	22.25%
Permanent hypocalcemia	0.00%	4.23%	5.56%	3.66%
Total	100%	100%	100%	100%

Finally, among a total of 355 patients, only 4 required reoperation for postoperative hemorrhage, representing 1.13% of cases.

## Discussion

Two controversial aspects emerge from the literature data:

- Lack of a Unified Definition for Retrosternal Disease: There is no universally accepted definition of what constitutes retrosternal pathology. Consequently, the reported incidence rates of retrosternal goiter vary widely across different studies, ranging from 2% to 26% (17).
- Predictive Factors for Additional Surgical Maneuvers: Although no definitive predictive factors are currently available to indicate the need for additional surgical maneuvers beyond the standard Kocher cervical incision, the use of secondary-level imaging and existing classification systems can be valuable in preoperative planning.

Surgical treatment remains the preferred approach for this condition (18).

At the General Surgery Department of Maria Vittoria Hospital, between January 1, 2014, and December 31, 2024, 355 cases of patients with thyroid pathology defined as “retrosternal” were identified, accounting for 26.4% of all thyroid surgeries performed during this period.

Of these, 84.2% (299 patients) had a preoperative diagnosis of multinodular goiter, consistent with the literature indicating that multinodular goiter is the most common thyroid pathology with retrosternal extension requiring surgical intervention (13).

Over the years, in line with the literature, we have observed an increasing utilization of secondary-level imaging to identify retrosternal thyroid pathology. The average usage rate rose from 23% in the first 6 years to 51.2% in the last 5 years.

The surgical approach employed at the Maria Vittoria Hospital General Surgery Unit aligns with established practices in the literature. The standard transcervical approach, specifically the classic Kocher incision, performed by an experienced team of endocrine surgeons, is sufficient to treat patients with retrosternal thyroid pathology.

At the reference center under study, among the 355 patients with retrosternal thyroid pathology who underwent surgical intervention, the classic Kocher cervical approach, with at most the use of split cutaneous incision, was employed in 99.2% of cases. In contrast, a thoracic approach was necessary in only 0.8% of cases, consistent with the findings reported in the literature.

Specifically, cervical incision was utilized in 317 cases (89.3%), cervical incision with split cutaneous approach in 35 cases (9.9%), sternotomy in 2 cases (0.6%), and a single case (0.2%) required posterolateral thoracotomy due to a large recurrent paratracheal right mediastinal goiter.

The data collected enabled the stratification of 128 patients who had undergone preoperative cervical CT or MRI imaging into the four classifications examined in this study. Analysis revealed that patients meeting the criteria of these classifications, or those falling into higher grades within scale-based classifications, indeed required a significantly higher percentage of extracervical surgical maneuvers compared to patients whose thyroid condition did not meet these criteria.

Notably, 55.56% of patients (5 out of 9) whose thyroid condition met Katlic’s definition required extended surgical maneuvers, compared to 15.13% of patients whose condition did not meet this definition, with a statistically significant difference (p-value = 0.002).

Utilization of Cohen and Cho’s classification allowed for a more granular stratification of the 128 patients, assigning them to the four grades defined by this system. As the grade increased, there was a statistically significant increase in the percentage of extracervical surgical maneuvers required (p-value = 0.003).

Among the 19 patients classified according to the definition of retrosternal extension as at least 3 cm behind the sternum, 9 (47.37%) required a surgical approach other than traditional cervical incision. In contrast, only 12.84% of patients whose condition did not meet this classification required additional surgical maneuvers, a statistically significant difference (p-value = 0.002).

Furthermore, eight patients had thyroid tissue extending to the level of the aortic arch (grade 2) or below it (grade 3). In these groups, 66.67% and 100% of patients, respectively, required extracervical surgical maneuvers. This difference was also statistically significant (p-value = 0.001).

These findings underscore that, while the majority of cases can be adequately managed with the standard cervical approach, the application of these classifications proves clinically useful in anticipating the complexity of the surgical approach and supporting more precise preoperative planning.

Numerous studies have reported that total thyroidectomy for retrosternal goiter is associated with higher complication rates compared to surgery performed for cervical goiter removal (19, 20). Specifically, patients undergoing surgery for retrosternal goiter have a higher likelihood of experiencing postoperative bleeding, transient and permanent unilateral or bilateral recurrent laryngeal nerve (RLN) injuries, transient hypoparathyroidism, and permanent hypoparathyroidism (21).

The literature describes a transient RLN palsy rate ranging from 0.5% to 18% and a permanent palsy rate between 0% and 5.8% for this patient population (22, 23). Conversely, the literature reports a higher risk of developing such complications in patients with thyroid neoplasia and those undergoing reintervention (16, 24).

Studies have observed that patients undergoing thyroidectomy for retrosternal goiter have a higher likelihood of developing transient hypoparathyroidism compared to those undergoing standard thyroidectomy (22). Indeed, the incidence of transient hypocalcemia after surgeries for retrosternal goiter ranges from 6.4% to 31.1%, with a permanent incidence around 4.1% (22).

Postoperative cervical hematoma presents an incidence rate ranging from 0.3% to 4.2% in cases of retrosternal thyroid disease (21).

The results obtained in the conducted study align with these reported percentages.

## Conclusion

In conclusion, total thyroidectomy for thyroid disease with retrosternal extension can be performed safely in the vast majority of cases through a cervical incision when surgery is carried out in high-volume centers with experienced surgical teams.

Computed tomography (CT) or magnetic resonance imaging (MRI) of the cervical region remain the modalities of choice for investigating the retrosternal component of the thyroid because they facilitate precise preoperative planning and assessment of mediastinal involvement. Although there is no universally accepted definition of substernal goitre in the literature, imaging-based criteria derived from second-level modalities have proven useful for classifying cases according to the degree of retrosternal extension, thereby supporting surgeons in selecting the most appropriate surgical approach.

In particular, in light of the findings of the present study, the integration of the Huins classification and the Cohen & Cho classification was demonstrated to be a valid predictive factor for the need to adopt a surgical approach beyond the classical Kocher-type cervicotomy. Specifically, patients classified as Grade 2 or 3 according to the Charles T. Huins classification, and those classified as at least Grade 2 according to the J.P. Cohen & H.T. Cho system, were more likely to require additional surgical maneuvers beyond the standard cervical incision (**Table 7**).

**Table 7:** Correlation between the Huins, Cohen & Cho classifications and surgical approach.

Definition	Cervicotomy	Extracervicotomy procedures	Total
Above the aortic arch	**104**	**16**	**120**
grade 1	80	10	90
grade 2	20	6	26
grade 3	3		3
grade 4	1		1
Between the aortic arch and the pericardium	**1**	**2**	**3**
grade 1	1		1
grade 2		2	2
Below the right atrium		**5**	**5**
grade 3		4	4
grade 4		1	1
**Total**	**105**	**23**	**128**

The bold values represent the totals for: above the aortic arch, at the level of the aortic arch, and below the aortic arch, respectively. The rows immediately below each of these indicate the breakdown according to the definition of Cohen and Cho. The last row represents the overall total.

Highlighted numbers show the patients classified as Grade 2 or 3 according to the Charles T. Huins classification (between the aortic arch and the pericardium – below the right atrium), and those classified as at least Grade 2 according to the J.P. Cohen & H.T. Cho system, were more likely to require additional surgical maneuvers beyond the standard cervical incision.

Our findings underscore that the necessity of an extracervical surgical approach, as well as the incidence of surgical complications, are heavily influenced by the experience of the surgical team.

On this basis, we strongly recommend that thyroid disorders with retrosternal extension be managed in high-volume referral centers equipped to provide the multidisciplinary expertise and specialised surgical experience required.

## Data Availability

The original contributions presented in the study are included in the article/supplementary material. Further inquiries can be directed to the corresponding authors.
